# Antidiabetic Properties and Toxicological Assessment of *Antidesma celebicum* Miq: Ethanolic Leaves Extract in Sprague–Dawley Rats

**DOI:** 10.1155/2022/2584698

**Published:** 2022-05-24

**Authors:** Raysa Y. Pratiwi, Berna Elya, Heri Setiawan, Roshamur C. Forestrania, Rizna T. Dewi

**Affiliations:** ^1^Faculty of Pharmacy, Universitas Indonesia, Depok, Indonesia; ^2^Research Center for Chemistry, Indonesian Institute of Sciences, Serpong, Indonesia

## Abstract

*Antidesma* is a genus of plants, and its several species are known to have antidiabetic properties. Leaves of Kayu Tuah (*Antidesma celebicum* Miq) have been proven to have the best *α*-glucosidase inhibition ability compared to other species in the *Antidesma* genus, as evidenced by the in vitro *α*-glucosidase inhibition test. However, no scientific studies have reported its antidiabetic properties and toxicity in vivo. Therefore, this research managed to verify the antidiabetic features and safety of ethanolic extract of *A. celebicum* leaves (EEAC) in Sprague–Dawley rats. Male rats (170–280 g) were induced diabetic with streptozotocin (35 mg/kg BW) and fed a high-fat diet comprising 24% fat, whereas control group rats were given a standard diet. Rats were treated with EEAC at 200 and 400 mg/kg BW doses for 28-days and 60 mg/kg BW acarbose for the control group. Determination of antidiabetic properties was done by analyzing lipid profiles as well as fasting blood glucose. After confirming the antidiabetic properties of EEAC, the toxicological assessment was determined using the fixed-dose method. General behavior changes, appearance, signs of toxicity, mortality, and body weight of animals were marked down during the observation period. When the treatment period ended, hematological, biochemical, and histological examinations of liver, kidneys, and heart sections were performed. The results confirmed that EEAC reduced fasting blood glucose levels and stepped forward lipid profiles of rats. Also, all animals survived, and no obvious destructive outcomes were noticeable during the study. As EEAC has promising results toward hyperglycemia and hyperlipidemia and has been proven safe through toxicity tests, it can be concluded that EEAC has good potential to be further developed into antidiabetic drugs.

## 1. Introduction

Diabetes is a worldwide health problem that maintains to grow globally. Both women and men are susceptible to developing diabetes. Women have a higher threat of developing diabetes in youth, even as men have a higher risk of developing diabetes in midlife. However, there is no considerable distinction in the prevalence or incidence of diabetes due to sex differences in people aged 20–49 years [1]. Diabetes mellitus is also known as a significant risk factor for severe COVID-19 disease. Moreover, based on experiments on pancreatic cell cultures, Sars-Cov-2 is known to trigger diabetes by damaging the cells that control blood glucose in the pancreas and liver [2].

Asia is one of the epicentres of the global diabetes pandemic, contributing to more than 60% of the world's diabetic population [3]. Despite modern advances in prevention and antidiabetic therapy, alternative treatment strategy using medicinal plants is still in demand, especially in Asia and Africa. Medicinal plants have also been widely embraced in the United Kingdom, Europe, Australia, and America [4].

Medicinal plants of *Antidesma* genus have been used for antidiabetic treatment. One of its species, *Antidesma madagascariense* Lam. is commonly used as a herbal remedy to deal with diabetes in Mascarene Islands, Madagascar [5]. Another species of *Antidesma* genus known as bignai in Filipino, *Antidesma bunius* is also used as a traditional antidiabetic medicine in the Philippines [6]. In Indonesia, there are also many plants of the *Antidesma* genus, such as *A. bunius*, *Antidesma neurocarpum*, *Antidesma montanum*, *Antidesma baccatum*, and *Antidesma celebicum*, which mostly grow in Jember, Sulawesi, Halmahera, and Ayawasi, Irian Jaya [7]. The results of screening research on *α*-glucosidase inhibitory activity in numerous plants, including the *Antidesma* confirmed that the ethanolic extract of the leaves of *A. celebicum* Miq. (AC), known as Kayu Tuah in Indonesian, had the prime *α*-glucosidase inhibitory activity. The investigation stated that the ethanolic extract from the leaves of this plant had a higher *α*-glucosidase inhibitory pastime than the control, acarbose [8]. It indicates that AC has the potential to be developed into herbal medicines that have antidiabetic properties.

Ethanolic extract of *A. celebicum leaves* (EEAC) is known to contain flavonoids, tannins, saponins, and glycosides [9]. Novi et al. [10] succeeded in isolating several active compounds from AC leaves that had *α*-glucosidase inhibitory activity, namely gallic acid; 3, 4, 5-trihydroxy-1-methoxybenzene; dioctyl phthalates; and dienoic acid. In vitro testing of the four compounds' activity against *α*-glucosidase inhibition showed an IC value of 0.057, 0.077, 0.206, and 0.182 mM, respectively, while the comparison control, acarbose, showed an IC50 value of 0.005 mM. Hence, it can be concluded that gallic acid compound is the most active content of AC leaves in *α*-glucosidase inhibitory activity.

Based on these studies, AC shows the potential to be developed into an antidiabetic drug. However, no scientific studies have reported its antidiabetic properties and toxicity. Therefore, this study aims to conduct an in vivo study regarding antidiabetic properties and the safety of EEAC against Sprague-Dawley rats.

## 2. Materials and Methods

### 2.1. Drugs and Chemicals

All the solvents used in this experiment were of analytical grade; these were ethanol, methanol, and ethyl acetate. Hydrochloric acid 37%, sulfuric acid 95–97%, acetic acid (glacial) 100%, sodium chloride, formaldehyde solution 37%, sodium hydroxide, citric acid monohydrate, sodium citrate monobasic ≥99.5% (Sigma-Aldrich, Germany), Folin–Ciocalteu's phenol reagent, Molisch reagent, magnesium powder, aluminum chloride, potassium iodide, iodine, benzene, and gallic acid. All chemicals used, unless otherwise stated, were obtained from the Merck Chemical Company (Darmstadt, Germany). Acarbose was generously provided by Dexa Medica, Indonesia, water for injection (Ikapharmindo Putramas, Indonesia), carboxymethyl cellulose (Brataco, Indonesia), streptozotocin (Wako, Fujifilm, Japan), xylazine (Interchemie, Netherland), and ketamine (Kepro, Netherland).

### 2.2. Preparation and Extraction

Leaves of AC received from Bogor Botanical Gardens, West Java, Indonesia, and identified by the Research Centre for Plant Conservation, Indonesian Institute of Sciences, Bogor, West Java, Indonesia (B.1015/IPH.3/KS/IX/2020). The leaves of AC were dried and powdered. EEAC was set by extracting 2 kg of powdered AC with 70% ethanol. The resulting filtrate was gathered and then evaporated using a rotary evaporator (Rotavapor®, Buchi, Switzerland) until a thick extract was obtained. The thick extract was then dried by the oven-drying (UN110, Memmert, Schwabach, Germany) method for two days at 50°C to get it dry. The % yield of EEAC was 16.7%.

### 2.3. Phytochemistry Test

Phytochemical identification tests conducted in this study consisted of alkaloid test with Bouchardat reagent, flavonoid test with aluminum complexation reaction, tannin test with gelatine test, saponin test with honeycomb froth test, terpenoid test with Lieberman–Bouchard reagent, anthraquinone test with Borntrager reaction, and glycoside test with Molisch reaction [8].

### 2.4. Estimation of Total Phenolic Content

Measurement of total phenolic content was carried out using the Folin–Ciocalteu technique with gallic acid as a standard. The primary solution of gallic acid was made with a concentration of 1000 ppm and then carried out on a series of dilutions with 10, 20, 40, 60, and 80 ppm, respectively. The extract was dissolved in methanol, and the concentration was determined to be 100 ppm. In 1 ml of the test solution and the standard solution series, each was poured into a suitable container, added 5 ml of Folin–Ciocalteu (7.5% in distilled water) and left at room temperature for 8 minutes. Then pour 4 ml of 1% NaOH and incubate for 1 hour. The absorption of each solution was measured using a spectrophotometer (T80+ UV/VIS Spectrometer, PG Instruments Ltd., Leicestershire, United Kingdom) with an absorption wavelength of 730 nm. Results are expressed as mg/g gallic acid equivalent (GAE) [11].

### 2.5. Animals Preparation

Male and female Sprague–Dawley (SD) rats (8–12 weeks old, 170–280 g) were purchased from the National Agency for Drug and Food Control. Rats had been acclimatized with free access to food and water for 10 days. They were kept under constant temperature (22 ± 3°C), humidity (70 ± 10%), and light-darkish cycle (12 h/12 h). Animal handling was done in line with the procedures outlined by the National Agency of Drug and Food Control of Indonesia No. 7 of 2014 [12].

### 2.6. Induction of Diabetes

Five rats were randomly selected to be in the normal control (NC) group and fed a standard feed (ND); 20 other rats received a high-fat diet (HFD) comprising 24% fat [13]. After 28 days of nutrition manipulation, diabetes was induced at day 29 through intraperitoneal injection of low-dose streptozotocin (STZ) dissolved in 0.1 M citrate buffer (pH 4.5, 35 mg/kg BW) [14]. Rats had fasted four hours earlier before STZ administration. The rats' feed was returned right away to the animals after the administration of STZ. The STZ-induced rats with the symptoms of diabetes and fasting blood glucose (FBG) levels of ≥200 mg/dL were decided on as diabetic models [15].

#### 2.6.1. Antidiabetic Activity Test Procedure

Twenty-five rats were divided into 5 groups, consisting of 5 rats each, as demonstrated:

1. Normal control (NC) rats.
2. Diabetic control (DC) rats.
3. DC + acarbose (60 mg/kg BW per day).
4. DC + EEAC (200 mg/kg BW per day).
5. DC + EEAC (400 mg/kg BW per day).

The examined samples were administered orally. Normal and diabetic controls received Na CMC aqueous solutions with similar volume (1 ml). The administration of extracts was persisted for 28 days, once a day.

#### 2.6.2. Biochemical Analysis

Blood samples were taken to quantify blood glucose and lipid profiles. Fasting blood glucose was measured after rats fasted for nine hours [16]. Blood drop was dripped out from the distal stop of the tail, carried out to a check strip, and analyzed at once using a blood glucose tracking device (AccuChek® Active, Roche, Germany) [17]. Blood samples were collected before and after STZ induction and at the end of the study for lipid profiles evaluation.

### 2.7. Acute Oral Toxicity

Forty rats (20 rats of each sex) were randomly placed into four groups (5 rats/sex/group; *n* = 10). The test was carried out using the fixed-dose method according to the Organization for Economic Co-operation and Development (OECD) Guideline No. 420 [18]. The acute oral toxicity test was carried out in a stepwise procedure with fixed doses of 5, 50, 300, 2000, and 5000 mg/kg BW extract. Under OECD Guideline No. 420, 300 mg/kg BW should be a starting dose for acute toxicity study when toxicological data of test substance is not available or limited. Animals may be dosed at a higher or lower fixed-dose relying on the presence or absence of toxicity symptoms or mortality [19].

#### 2.7.1. Body and Organ Weight Assessment

Rats' bodyweight was well noted on the day before the acute toxicity test and after the test was done (day 0 and 15). The liver, kidneys, spleen, heart, and lung were isolated and weighed after the gross necropsy. Relative organ weight was then quantified by dividing organ weight by body weight [20].

#### 2.7.2. Hematological and Biochemical Analysis

Hematological analyzer, BC 2800 (Mindray Bio-Medical Electronics Co. Ltd., Shenzhen, China) was used to perform hematological analysis. The examination parameters included red blood cells (RBC), hemoglobin (HGB), hematocrit (HCT), mean cell volume (MCV), mean cell hemoglobin (MCH), mean cell hemoglobin concentration (MCHC), and white blood cells (WBC). Biochemical analysis was performed using a DumoChem 20 (DUMO, Austria) to measure and assess levels of aspartate transferase (AST), alanine transaminase (ALT), urea, and creatinine.

#### 2.7.3. Histopathological Analysis

Tissues from the liver, kidneys, and heart were isolated for histological investigation. The tissues were placed in 10% neutral buffered formalin. Hematoxylin-eosin staining was used to analyze and evaluate the tissues' histological condition beneath the light microscope (Olympus BX51, Japan) [21].

### 2.8. Statistical Analysis

Data are presented as the mean values ± standard error the mean (SEM) and statistically analyzed using GraphPad Prism version 8. The acquired data were analyzed for normality and homogeneity of variance by the Shapiro–Wilk and Levene tests. Normally distributed and homogenous data were further analyzed by the one-way variance (ANOVA) analysis followed by the Tukey post hoc test. Meanwhile, nonnormally distributed and heterogeneous data were analyzed by the Kruskal–Wallis test and the Dunn's Multiple Comparison Test. Mean difference with *p*-value <0.05 was considered significant [22].

## 3. Results and Discussion

Phytochemical analysis of EEAC was carried out as a first step in searching for active ingredients responsible for biological activity. Moreover, it provided a basis for the isolation of the targeted compound and may lead to the discovery and development of drugs [23]. Qualitative phytochemical analysis of EEAC confirmed the presence of various substances, such as flavonoid, tannin, saponin, anthraquinone, and glycoside.

This study detected flavonoid, which is a part of hydroxylated phenolic substances. These results were also in line with the previous study, which analyzed the phytochemical content of EEAC extracted using the reflux method [8]. Flavonoids are potent antioxidants and have attracted activity against free radical-mediated diseases, particularly diabetes mellitus, as some studies have shown that antioxidants can improve insulin action. In diabetic conditions (particularly type 2 diabetes), hyperglycemia, hyperlipidemia, and elevated inflammatory cytokines are caused by insulin resistance and inadequate glucose-induced insulin secretion. This condition causes oxidative stress, which triggers cells death, including pancreatic *β*-cells. Many studies, both in vivo and in vitro, have proven that flavonoids can protect against *β*-cell degeneration caused by diabetes. Oxidative stress can be suppressed because flavonoids are able to inhibit the accumulation of ROS and lipid peroxidase, as well as inhibit DNA damage by increasing Bcl-2 expression and inhibiting proapoptotic gene expression [24]. A study stated that one type of flavonoid, namely rutin, can increase the expression of PPAR*γ*. PPAR*γ* is a gene that has a regulatory effect on the transcription of PDX-1, which regulates the development and subsequent differentiation of *β*-cells. Under conditions of increased PPAR*γ* expression, PDX-1 expression will also increase. This condition promotes the regeneration and proliferation of *β*-cells, which lead to the improvement of diabetic conditions [25, 26].

**Figure 1:**
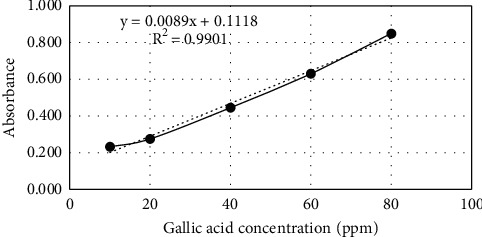
Calibration curve.

Total phenol content was carried out to determine the approximate amount of phenol content in EEAC.  Table 1 presents the gallic acid absorbance, and the calibration curve is shown in Figure 1. The linear regression equation was (*y*) = 0.0089*x* + 0.1118 with a value of *r*^2^ = 0.9901. Calculation of the total phenolic content was obtained by substituting the EEAC (*y*) absorbance value into the linear regression equation obtained from the gallic acid calibration curve. From the measurement, the absorbance of EEAC (*y*) was 0.278. It can be concluded that the total phenol content in EEAC was 18.712 ppm or 187.12 g GAE [27]. EEAC is known to have an antidiabetic effect by inhibiting *α*-glucosidase activity mainly due to its phenolic content (i.e., gallic acid) [10]. Phenols can control starch digestion by forming hydrogen bonds with polar digestive enzymes such as *α*-amylase and *α*-glucosidase. The effect of this interaction can inhibit the action of enzymes, thereby inhibiting glucose absorption and preventing hyperglycemia [28].

The most significant weight gain was seen in the NC group, which could be attributed to normal insulin levels (Table 2). The NC group did not experience pancreatic damage due to STZ administration; therefore, insulin in the NC group works optimally to store excess nutrition as glycogen and fat in several tissues. Lack of insulin in rats can trigger the loss of adipose tissue where fat is stored, making it difficult to gain weight [29]. In addition, diabetic rats generally experience physiological and psychological stress such as increased thirst, excitable urination, severe hunger, discomfort, and fatigue, making it difficult to gain weight [30]. In this study, the bodyweight of all diabetic rats moderately increased compared to the NC group. Administration of EEAC induced more weight gain in a manner similar to acarbose. It indicated that the extract could relieve the physiological and psychological stress experienced by diabetic animals.

Table 3 presents the FBG levels. FBG levels of diabetic rats were considerably higher than those of control rats on day 0 and 28. FBG in the acarbose-treated group decreased significantly at day 28. Zhang et al. discovered that acarbose could modify glucose metabolism through the MAPK pathway and suppress proinflammatory cytokines by increasing miR-10a-5p and miR-664 within the ileum. Thus, acarbose as positive control is considered suitable [31].

Furthermore, EEAC is known to have *α*-glucosidase inhibitory activity whose mechanism of action is similar to that of acarbose [10]. Oral administration of EEAC for 28 days drastically reduced blood glucose level compared to the diabetic control group, and this reduction in FBG level was similar to the acarbose-treated group. However, all treated groups have significant FBG levels compared to the normal control. Further investigation is needed to determine whether or not the extract can normalize FBG level if administered over an extra prolonged length and the way the extract promises this impact at a molecular degree.

The EEAC doses selected for this antidiabetic activity study were 200 and 400 mg/kg. This dose was selected referring to the results of previous studies regarding the antidiabetic activity of plants in the same genus as *A. celebicum*, namely *A. bunius*. It was found that the administration of *A. bunius* extract at a dose of 250 mg/kg BW for 28 days and 500 mg/kg BW for 14 days ought to reduce rat blood glucose. It is hoped that by giving the same dose as in the *A. bunius* study, EEAC will also provide similar benefits [6, 32]. When translated into human doses, the amount of 200 mg/kg BW in rats is equal to 37.84 mg/kg BW in humans, while a dose of 400 mg/kg BW is equal to 75.68 mg/kg BW; this means it takes 2–4.5 g of EEAC for a human weighing 60 kg [33]. Further study is needed to decide the most advantageous dose of EEAC that could offer the expected antidiabetic activity.

The levels of rats' lipid profiles after the 28-day EEAC administration are shown in Table 4. It showed that the diabetic condition causes a noticeable increase in several lipid profile parameters. Administration of EEAC for 28 days could normalize cholesterol, LDL, and triglyceride levels similar to acarbose treatment. However, acarbose treatment significantly lowered triglyceride levels compared to EEAC treatment. Diabetes is generally accompanied by dyslipidemia due to insufficient insulin, which contributes to the regulation of lipid metabolism. Insulin promotes glucose access into adipocytes, and glucose may be used to synthesize glycerol inside those cells. The glycerol and the fatty acids brought from the liver synthesize triglycerides within the adipocyte. These mechanisms involve insulin in the further accumulation of triglycerides in fat cells. Furthermore, glucose and fatty acids cannot be stored in the tissues if insufficient insulin occurs, resulting in dyslipidemia [34]. EEAC 400 mg/kg orally showed a better effect in normalizing lipid profiles than EEAC 200 mg/kg, and this may be due to the presence of phenols that lower lipids in the blood by enhancing its storage via the insulin signaling pathway and, therefore, blood lipid levels can be suppressed [35].

Streptozotocin-induced diabetes is known to cause liver damage. Therefore, examination of liver function parameters (e.g., AST and ALT) and liver histopathological examination should be considered to see liver function after STZ induction and its improvement after treatment and ensures that changes in lipid profile are not caused by damage to the liver [36].

After obtaining adequate data regarding the antidiabetic effect of EEAC, it is necessary to evaluate the safety of EEAC. Administration of EEAC at several levels of fixed-dose doses certainly had no impact on clinical symptoms. All rats presented similar appearance and behavior throughout the observation period. No deaths were seen in any of the groups during the study period. As shown in Table 5, the extract had no significant impact on rats' body weight changes. All rats experienced moderate gain weight, indicating that the rats were in good health [30].

Rat relative organ weights were calculated as shown in Table 6. Since essential organs, along with the liver, kidneys, heart, lungs, and spleen, are functionally vital organs often impaired with the aid of toxic substances [19], rough examinations of internal organs were performed to identify potential signs of organ-targeted toxicity. The relative heart weight in the female group receiving a 300 mg/kg dose appeared smaller than in the other groups. De Carvalho and Thomazini [37] observed that relative heart weight on female rats is 0.29 ± 0.015, so this difference was still within the normal range. Relative organ weight is strongly influenced by rats' age, sex, and body weight [37]. Moreover, no significant effects were visible on rats' relative organ weight, and no lesions were discovered upon macroscopic examination.

The overall fitness status of the rats was also examined by hematological and biochemical evaluation. Except for urea that confirmed a widespread increase, all other biochemical and hematological assessment parameters remained unaffected by the extract (Table 7). Nevertheless, these changes had been inside the regular reference variety for rats. Rats urea is resistant to age and gender-related variation, and it is within the scope of 32–48 mg/dl [38]. Urea is one of the conventional biomarkers commonly used to detect nephrotoxicity. However, due to its low sensitivity, it is necessary to examine other biomarkers, for example, creatinine, aspartate aminotransferase (AST), lactate dehydrogenase (LDH), N-acetyl-*β*-D-glucosaminidase (NAG), and accompanied by renal histopathological examination [39]. In this study, creatinine, AST, and renal histopathology results were normal, so it can be concluded that the increase in urea levels in rats may be caused by the high consumption of dietary protein [40]. Investigation of HE-stained liver, kidneys, and heart sections revealed the typical architecture of normal organs. No adverse histopathological presentations were observed in all control and treatment groups.

## 4. Conclusion

EEAC has promising effects on hyperglycemia and hyperlipidemia. Administration of EEAC 400 mg/kg orally showed the best outcome in decreasing FBG level and normalizing lipid profiles compared to EEAC 200 mg/kg. The acute oral toxicity study of EEAC revealed its safety. The results confirmed no noticeable differences in clinical symptoms, body weight changes, relative organ weight, hematological and biochemical parameters, organ dissection, and histological presentations in comparison to the control group. Taking these results together, it can be concluded that EEAC has good potential to be further developed into antidiabetic drugs.

## Figures and Tables

**Table 1:** Gallic acid absorbance.

Concentration (ppm)	Absorbance
10	0.232
20	0.275
40	0.445
60	0.630
80	0.849

**Table 2:** Body weight of experimental animals after 28 days oral administration of EEAC from antidiabetic activity study.

Groups	Body weight (g)		
	Day 0	Day 28	Weight gain
NC	250.80 ± 5.76	363.00 ± 8.98^bc^	112.20 ± 11.48^b^
DC	231.80 ± 13.89	245.90 ± 26.85^a^	14.02 ± 19.09^a^
DC + 200 mg/kg EEAC	225.40 ± 6.28	263.50 ± 12.00^a^	38.10 ± 10.64
DC + 400 mg/kg EEAC	219.10 ± 17.92	261.40 ± 21.26^a^	42.26 ± 9.85
DC + acarbose	207.40 ± 9.74	271.90 ± 3.27^a^	64.44 ± 11.61

NC: normal control, DC: diabetic control. Results are expressed as mean ± SEM (*n* = 5). (^a^) means significantly different from NC group; (^b^) means significantly different from DC group; (^c^) means significantly different from DC + acarbose group (*p* < 0.05).

**Table 3:** Fasting blood glucose of experimental animals after 28 days oral administration of EEAC from antidiabetic activity study.

Groups	FBG (mg/dl)	
	Day 0	Day 28
NC	89.80 ± 2.82^bc^	95.00 ± 1.52^bc^
DC	358.40 ± 17.10^a^	515.60 ± 29.37^ac^
DC + 200 mg/kg EEAC	366.80 ± 21.88^a^	332.80 ± 12.88^ab^
DC + 400 mg/kg EEAC	346.20 ± 18.50^a^	252.00 ± 23.18^ab^
DC + acarbose	359.80 ± 18.70^a^	244.00 ± 35.39^ab^

NC: normal control, DC: diabetic control. Results are expressed as mean ± SEM (*n* = 5). (^a^) means significantly different from NC group; (^b^) means significantly different from DC group; (^c^) means significantly different from DC + acarbose group (*p* < 0.05).

**Table 4:** Lipid profiles of experimental animals after 28 days oral administration of EEAC from antidiabetic activity study.

Groups	Lipid profiles (mg/dl)			
	CHOL	HDL	LDL	TG
NC	61.60 ± 3.14^bd^	35.30 ± 1.29	37.20 ± 0.80^b^	93.30 ± 4.14^b^
DC	144.60 ± 19.47^ac^	43.94 ± 3.55	61.40 ± 5.35^a^	269.40 ± 69.29^a^
DC + 200 mg/kg EEAC	138.00 ± 14.71^a^	43.10 ± 5.13	60.40 ± 3.14^a^	253.80 ± 15.57^a^
DC + 400 mg/kg EEAC	79.20 ± 7.91^bd^	39.70 ± 4.50	45.00 ± 5.22	182.40 ± 25.63
DC + acarbose	87.00 ± 8.60^b^	39.76 ± 3.65	47.20 ± 3.65	130.20 ± 12.46

NC: normal control, DC: diabetic control, CHOL: cholesterol, HDL: high-density lipoprotein, LDL: low-density lipoprotein, TG: triglyceride. Results are expressed as mean ± SEM (*n* = 5). (^a^) means significantly different from NC group; (^b^) means significantly different from DC group; (^c^) means significantly different from DC + acarbose group; (^d^) means significantly different from DC + 200 mg/kg EEAC group (*p* < 0.05).

**Table 5:** Body weight changes of experimental animals after 14 days of single oral administration of different doses of EEAC from acute toxicity study.

Sex	Dose (mg/kg BW)	Weight gain (g)
Male	0	77.10 ± 5.98
	300	60.52 ± 8.77
	2000	51.72 ± 5.84
	5000	55.78 ± 9.80

Female	0	29.36 ± 2.85
	300	36.56 ± 3.88
	2000	31.54 ± 1.70
	5000	39.22 ± 4.83

Data are expressed as Mean ± SEM (*n* = 5). Significantly different from control group was designated as ^*∗*^(*p* < 0.05).

**Table 6:** Relative organ weight of experimental animals after 14 days of single oral administration of different doses of EEAC from acute toxicity study.

Sex	Dose (mg/kg BW)	Relative organ weight (g/kg)					
		Liver	Kidney (R)	Kidney (L)	Heart	Spleen	Lung
Male	0	35.98 ± 0.96	3.55 ± 0.12	3.80 ± 0.13	3.19 ± 0.15	2.23 ± 0.09	4.99 ± 0.23
	300	35.34 ± 3.41	3.68 ± 0.42	3.70 ± 0.48	3.17 ± 0.42	2.58 ± 0.14	6.64 ± 0.51
	2000	34.10 ± 1.25	4.08 ± 0.18	4.08 ± 0.18	3.03 ± 0.11	2.74 ± 0.15	6.61 ± 0.33
	5000	35.26 ± 1.96	3.42 ± 0.25	3.39 ± 0.22	3.19 ± 0.21	2.56 ± 0.13	6.44 ± 0.95

Female	0	36.35 ± 2.32	3.47 ± 0.21	3.55 ± 0.16	3.46 ± 0.05	2.45 ± 0.07	5.66 ± 0.31
	300	33.43 ± 2.47	3.25 ± 0.30	3.25 ± 0.30	2.72 ± 0.29^*∗*^	2.53 ± 0.18	5.84 ± 0.54
	2000	37.38 ± 1.73	3.27 ± 0.24	3.45 ± 0.26	2.91 ± 0.16	2.67 ± 0.18	6.56 ± 0.83
	5000	33.80 ± 2.79	3.60 ± 0.20	3.69 ± 0.17	2.99 ± 0.10	2.67 ± 0.18	9.98 ± 3.36

Data are expressed as Mean ± SEM (*n* = 5). Significantly different from control group was designated as ^*∗*^(*p* < 0.05).

**Table 7:** Hematological and biochemical values for rats treated with EEAC in acute toxicity study.

Parameter	Dose (mg/kg BW)			
	0	300	2000	5000
Male				
RBC (×10^6^/*µ*l)	7.10 ± 0.38	7.56 ± 0.28	7.42 ± 0.20	7.70 ± 0.38
HGB (mg/dl)	13.98 ± 0.55	14.68 ± 0.41	14.64 ± 0.50	14.56 ± 0.58
Hematocrit (%)	38.20 ± 1.59	42.60 ± 1.25	41.20 ± 0.97	40.80 ± 1.20
MCV (fl)	54.10 ± 0.61	56.40 ± 1.86	55.40 ± 0.98	53.40 ± 1.78
MCH (pg)	19.82 ± 0.39	19.40 ± 0.51	19.60 ± 0.24	18.80 ± 0.49
MCHC (g/dl)	36.64 ± 0.35	34.40 ± 0.68	34.80 ± 0.86	35.60 ± 0.68
WBC (×10^3^/*µ*l)	7.38 ± 0.48	7.94 ± 0.87	8.52 ± 0.79	7.42 ± 1.12
AST (I/U)	156.20 ± 3.80	136.20 ± 12.76	200.2 ± 33.98	172.00 ± 20.08
ALT (I/U)	64.28 ± 6.38	55.80 ± 4.18	59.00 ± 3.62	54.00 ± 6.67
Urea (mg/dl)	32.80 ± 3.52	48.72 ± 1.62^*∗*^	43.56 ± 1.95	48.14 ± 2.35^*∗*^
Creatinine (mg/dl)	0.52 ± 0.05	0.68 ± 0.04	0.64 ± 0.03	0.62 ± 0.04

Female				
RBC (×10^6^/*µ*l)	6.84 ± 0.24	6.84 ± 0.23	6.32 ± 0.31	6.94 ± 0.29
HGB (mg/dl)	13.76 ± 0.59	13.74 ± 0.34	12.80 ± 0.40	13.62 ± 0.36
Hematocrit (%)	38.60 ± 1.81	37.60 ± 1.29	36.40 ± 1.60	39.00 ± 1.18
MCV (fl)	56.18 ± 0.66	54.80 ± 1.39	58.00 ± 2.37	56.20 ± 1.20
MCH (pg)	20.10 ± 0.41	20.20 ± 0.49	20.40 ± 0.75	19.60 ± 0.40
MCHC (g/dl)	35.80 ± 0.48	37.00 ± 0.77	35.20 ± 0.58	35.20 ± 0.20
WBC (×10^3^/*µ*l)	5.82 ± 0.30	6.84 ± 0.76	6.82 ± 0.72	5.46 ± 0.61
AST (I/U)	103.60 ± 9.63	141.80 ± 16.30	96.40 ± 2.21	111.60 ± 16.92
ALT (I/U)	49.92 ± 11.55	54.80 ± 4.55	48.60 ± 2.68	47.40 ± 2.86
Urea (mg/dl)	30.60 ± 1.65	43.92 ± 0.76^*∗*^	45.04 ± 1.72^*∗*^	41.46 ± 4.22
Creatinine (mg/dl)	0.64 ± 0.09	0.80 ± 0.03	0.60 ± 0.03	0.66 ± 0.04

RBC: red blood cell count, HGB: hemoglobin, MCV: mean corpuscular volume, MCH: mean corpuscular hemoglobin, MCHC: mean corpuscular hemoglobin concentration, WBC: white blood cell count, AST: aspartate aminotransferase, ALT: alanine aminotransferase. Data are means ± SEM for groups of five rats/sex/dose ^*∗*^(*p* < 0.05) vs. control group.

## Data Availability

The data used to support the ﬁndings of this study are included within the article.
